# Estimating Partnership Duration among MSM in Belgium—A Modeling Study

**DOI:** 10.3390/idr16030032

**Published:** 2024-05-06

**Authors:** Achilleas Tsoumanis, Wim Vanden Berghe, Niel Hens, Christophe Van Dijck

**Affiliations:** 1 Department of Clinical Sciences, Institute of Tropical Medicine Antwerp, Nationalestraat 155, 2000 Antwerp, Belgium; wvandenberghe@itg.be (W.V.B.);; 2 Centre for Health Economics Research and Modelling Infectious Diseases (CHERMID), Vaccine and Infectious Disease Institute (VAXINFECTIO), University of Antwerp, 2610 Antwerp, Belgium; niel.hens@uhasselt.be; 3 Interuniversity Institute for Biostatistics and Statistical Bioinformatics, Data Science Institute, Hasselt University, 3590 Diepenbeek, Belgium

**Keywords:** sexually transmitted diseases, men who have sex with men, mathematical model, mathematical modeling, partnership duration, homophily

## Abstract

Mathematical modeling is widely used for describing infection transmission and evaluating interventions. The lack of reliable social parameters in the literature has been mentioned by many modeling studies, leading to limitations in the validity and interpretation of the results. Using data from the European MSM Internet survey 2017, we developed a network model to describe sex acts among MSM in Belgium. The model simulates daily sex acts among steady, persistent casual and one-off partners in a population of 10,000 MSM, grouped as low- or high-activity by using three different definitions. Model calibration was used to estimate partnership duration and homophily rates to match the distribution of cumulative sex partners over 12 months. We estimated an average duration between 1065 and 1409 days for steady partnerships, 4–6 and 251–299 days for assortative high- and low-activity individuals and 8–13 days for disassortative persistent casual partnerships, respectively, varying across the three definitions. High-quality data on social network and behavioral parameters are scarce in the literature. Our study addresses this lack of information by providing a method to estimate crucial parameters for network specification.

## 1. Introduction

The incidence of *Chlamydia trachomatis* (CT) and *Neisseria gonorrhoeae* (NG) is increasing in many European countries [1,2] and elsewhere [3,4], with a raising concern regarding NG, which has developed resistance to all classes of antibiotics it has been exposed to, including the currently recommended therapies [5,6,7]. Around half of the reported gonorrhea cases in European countries [1] (48%) and the US [3] (47%) are attributed to men who have sex with men (MSM).

Several modeling studies have described the transmission of CT and NG among MSM [8,9,10,11,12,13,14,15,16,17,18,19,20,21,22,23,24,25,26,27,28,29,30,31,32,33,34,35,36,37,38,39,40,41,42,43,44,45], which cluster in a few Western countries, mainly from the US [9,13,14,19,25,26,27,28,35,36] and Australia [18,22,23,24,32,40,41,42,43]. In Europe, notable modeling studies have been conducted in the UK [31,37,38,45] and The Netherlands [17,29,30,33,39,44], with few papers from France [34], Belgium [8,16] and Switzerland [20,21]. The majority of the published models from Europe are compartmental models, with only a hand-full of models using individual-based or network models [8,16,29,30,39,45]. While the usefulness of compartmental models is undeniable, they lack both the complexity of the transmission mechanisms of STIs and the fine-grain detail of individual characteristics that individual-based and network models exhibit.

Many models mention the lack of parameters in the literature to inform simulation models, leading to limitations in the validity and interpretation of the results [8,16,17,18,26,29,32,34,35,40,41,43,45]. Besides transmission probabilities that are difficult to calculate in a clinical setting, high-quality clinical parameters such as the probability of symptoms and time intervals until recovery are not readily available in the literature, especially when reported by different anatomical sites [46,47]. Another set of important parameters that are sparse in the literature are parameters critical to the structure of the sexual network, i.e., partnership duration and homophily rates. The role of homophily or the tendency of people to form sexual partnerships or other kinds of social bonds with persons similar to them in terms of various characteristics has been well established in the social network literature [48,49,50,51,52]. These parameters are vital for the validity of the results. If the underlying network is mis-specified, one cannot guarantee that the model behavior is due to the mechanisms included and their parameters, or due to the structure of the network. A limited number of cohort and network cross-sectional studies are cited in individual-based and network models, in terms of partnership duration and homophily rates in the US [53,54] and Australia [55]. In a European setting, estimates of partnership durations, came from a series of modeling papers from The Netherlands [29,30,39], where data from two Dutch studies were used to estimate the distributions of the duration of steady and casual partnerships. For the steady partnerships, a Weibull distribution was estimated with a shape parameter of 0.61 and scale of 920, based on data from the Amsterdam Cohort Study (ACS) among MSM [56]. Based on that, we estimated an expected mean duration for steady partnerships of 1355 days. For casual partnerships, a Gamma distribution with a shape of 0.03 and rate of 0.0002 was estimated based on the Network Study among MSM in Amsterdam data [57], yielding a mean duration of 155 days for casual partnerships.

Model calibration is widely used to estimate parameters that are difficult to find in the literature, i.e., transmission probabilities. In this paper, we aim to use model calibration to estimate the average partnership duration and homophily rates among MSM in Belgium in order to match the cumulative number of sex partners.

## 2. Methods

### 2.1. Data

Data from the Belgian-based participants of the European MSM Internet Survey (EMIS) 2017 were the primary data source for network structure and behavioral parameters used in the model. EMIS was an anonymous, self-administered online survey conducted in 50 countries and 33 languages. Participants were recruited through dating apps and other social networking websites targeting MSM (Romeo, Grindr, Hornet, Facebook, Twitter, Instagram). The methods of EMIS-2017 study have been described in detail elsewhere [58]. In total, questionnaire data from 2763 persons based in Belgium were used to estimate parameters, such as information on partnership status and behavioral and epidemiological characteristics.

### 2.2. Overview

We developed a network model to describe sex acts among MSM in Belgium. Separable Temporal Exponential-family Random Graph Models (STERGMs) [59,60,61] were used to fit and simulate the structure of the sexual partnership network. The model was developed as an extension of the EpiModel platform (www.epimodel.org, accessed on 15 October 2023).

Three different definitions for high sexual activity that were previously used in other modeling studies were included as scenarios in the model: (1) Eligibility to PrEP [16], (2) individuals reporting more than 15 partners [62] and (3) individuals reporting more than 15 casual partners [63]. Individuals in the population were categorized into high- and low-activity (HA-MSM and LA-MSM, respectively). In Belgium, eligibility for PrEP includes being above 18 years old, being HIV negative and fulfilling one of the following criteria: (1) engaging in anal sex without a condom with at least two partners in the previous 6 months, (2) having multiple sexually transmitted infections (STIs) in the previous 12 months, (3) taking post-exposure prophylaxis (PEP) multiple times in the previous 12 months or (4) using psychotropic substances (drugs) during sexual activities.

Based on the EMIS-2017 data, we estimated that 34.6% (32.87–36.45; 95% CI) of the Belgian MSM population would be eligible to receive Pre-Exposure Prophylaxis (PrEP). This estimate is similar to estimates from previously published studies from Belgium and Europe [62,64,65]. According to the other two definitions of high-activity MSM, 30.41% (28.69–32.19; 95% CI) and 27.90% (26.23–29.64; 95% CI) of the EMIS-2017 participants reported more than 15 total and casual partners over a 12-month period, respectively. Thus, 34.6%, 30.41% and 27.90% of the model population was classified as HA-MSM in the three scenarios, respectively, and the remaining was classified as LA-MSM.

The model consisted of three parallel, interacting networks representing steady, persistent casual and one-off (one-night stand) partnerships. The term “steady partners” was used to describe husbands, boyfriends or the partners with whom EMIS participants would not describe themselves as single. Persistent casual partners would be the partners that would not qualify as steady, but the EMIS participants would have sex with more than one time. One-off partners would be casual partners that EMIS participants would only have sex with once. All definitions, processes and parameters in the model are described in detail in Supplementary Materials.

### 2.3. Partnership Formation and Homophily

The formation of partnerships in all three networks (steady, casual and one-off) was governed by similar formation equations, in order to be able to preserve the distinct behavioral characteristics linked to each partnership type. The formation of steady (and casual) partnerships was associated with the total number of steady (casual) partnerships currently in the network, the proportion of concordant partnerships (HA-MSM with HA-MSM or LA-MSM with LA-MSM) which were different for each group, the proportion of individuals with concurrent partners (2 or more active partnerships simultaneously) and their status regarding casual (steady) partners (proportion of individuals with 0, 1 or more than 1 casual (steady) partners). The formation of one-off partnerships depended on the total number of one-off partnerships, the proportion of concordant partnerships (different for each group), the proportion of individuals with 0, 1 or more than 1 steady partners and the proportion of individuals with 0, 1 or more than 1 casual partners. All those characteristics were targeted to match observed statistics from the EMIS 2017 dataset for each group and partnership type and the underlying network was fitted so that they would be held constant over time.

The number of ongoing partnerships (steady and persistent casual) and their combination was estimated using the EMIS-2017 dataset. We allowed each individual in the model to have zero, one or more than one steady partner and zero, one or more than one casual partners at any given time step.

For both steady and persistent casual partnerships, there was a constant hazard of relationship dissolution, modeled as a memoryless process. For steady partnerships, we used a constant hazard depending on the total number of partnerships present in the network. For casual partners, the dissolution of partnerships depended on the activity group of the two partners in a dyad (different for the HA- and LA-MSM). The duration of one-off partnerships was set to 1 day.

### 2.4. Homophily

Regarding homophily, we adopted the premise from the paper of Hansson et al. [62] that the number of HA-individuals having LA-partners should be the same as the number of LA-individuals having a HA-partner within the same MSM population, to calculate the ratio of the homophily rates between the two groups for each type of partnership type. The process of deducting the final equations is shown in detail in Supplementary Materials.

### 2.5. Sex Acts

At each time step, the number of sexual acts that occurred between two partners was calculated by random draws from a Bernoulli distribution with a success probability depending on the type of partnership and the activity-group of the two partners. Each sex act could be a combination of six sex types: oral, oro-anal and anal sex, each of which could be insertive or receptive. For each sex act, a combination of sex types was randomly assigned based on the frequency reported among EMIS 2017 participants (Supplementary Materials Table S7).

### 2.6. Model Calibration

The model simulated a population of 10,000 MSM in Belgium. Parameters for partnership duration and homophily rates were not available in the EMIS data. We opted to use the cumulative number of partners per partnership type and group over a period of 12 months as a measure of goodness-of-fit for our model. Approximate Bayesian computation with sequential Monte Carlo (ABC-SMC) sampling [66,67,68,69] was used to estimate the unknown parameters. The ABC-SMC method returns posterior distributions of the parameters of interest after defining prior distributions. The Lenormand method of the EasyABC package [70] (Version 1.5, https://hal.inrae.fr/hal-04086912, accessed on 2 December 2023) was used for the parameter estimation.

## 3. Results

*Distribution of Cumulative Sex Partners*

The total numbers of steady and casual partners over a period of 12 months are reported in the EMIS 2017 dataset as categorical variables: 0, 1, 2, …, 9 or 10 or more for steady and 0, 1, 2, …, 10, 11–20, 21–30, 31–40, 41–50 or more than 50 for casual partners. Assuming at most 12 steady partners over 12 months, we used the methodology and statistics described by Mendez-Lopez et al. [71] to build an algorithm to assign integer values to the total number of partners that match the mean (standard deviation) and median (interquartile range) for each category level and overall (Table 1). At each iteration of the algorithm, we randomly assigned persons reporting more than 10 steady partners into the categories of 10, 11 or 12. For participants who had reported 10 or fewer casual partners, the sum of steady and casual partners was calculated. For those who reported more than 10 casual partners, the possible range of the sum of steady and casual partners was determined, and if the result spanned two EMIS categories (e.g., 11–20, 21–30, etc.), one of them was randomly selected with a probability based on the overlap of the two categories. Subsequently, random integers with the reported target mean and standard deviation were generated, and the resulting means and medians both by category and overall were compared to the target statistics. The algorithm iterated until an absolute sum of errors below 1.5 was produced. The produced statistics are presented in Table 1.

With the newly estimated number of total partners, we could calculate the cumulative number of steady and casual partners per risk-group over 12 months. The number of one-off partners was calculated by multiplying the total number of casual partners with the proportion of EMIS participants reporting a one-off partnership in their last sexual encounter (Supplementary Materials Table S4). The remaining casual partners were classified as persistent casual partners. The distributions of the cumulative number of partners per relationship type and group were summarized as medians and interquartile ranges, and were used as target statistics in the calibration process of the model.

**Figure 1:**
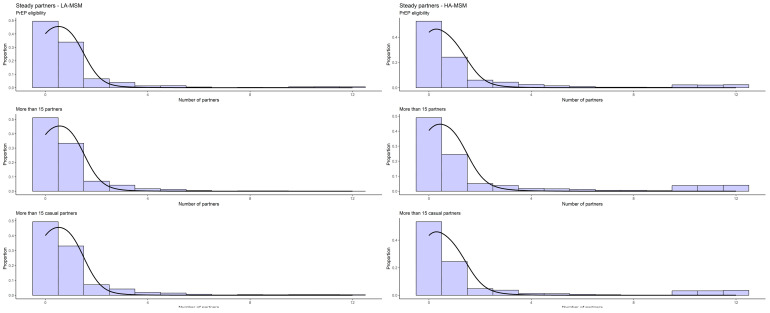
Observed (histogram) and simulated (line) cumulative sex partner distribution (12 months) among steady partnerships.

**Figure 2:**
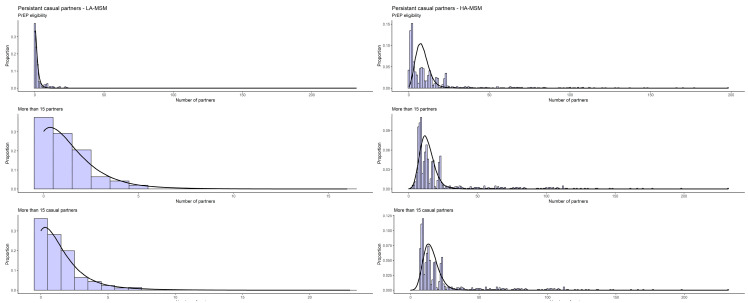
Observed (histogram) and simulated (line) cumulative sex partner distribution (12 months) among persistent casual partnerships.

**Figure 3:**
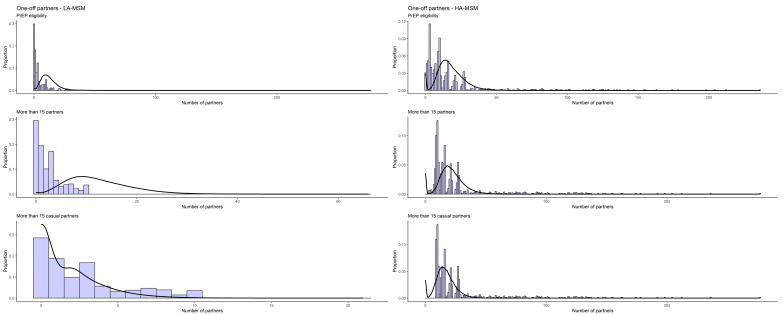
Observed (histogram) and simulated (line) cumulative sex partner distribution (12 months) among one-off casual partnerships.

The final estimates for partnership durations were between 1065 and 1409 days for steady, and 4–6, 251–299 and 8–13 days for assortative HA-, LA- and mixed persistent casual partnerships, respectively, varying across the three definitions for the activity group (Table 2). The estimated homophily rates for all three relationship types were high for steady and one-off partnership, for both activity groups, but lower for persistent casual partnerships (Table 2). The distributions of the observed and simulated cumulative number of partners in a period of 12 months is shown below as a histogram for the EMIS-reported partners and as a density plot for the simulated ones (Figure 1, Figure 2 and Figure 3).

## 4. Discussion

We estimated the average partnership durations among MSM in Belgium, distinguishing for the activity group, by employing three different definitions that have been used in previous modeling studies. Estimates for partnership durations and homophily rates were similar in all three scenarios, indicating quite robust results. The estimates for the duration of steady partnerships were comparable to the data reported by the ACS study [56] (1355 days), as well as studies from Hui et al. [22,23,24] and Kasaie et al. [26], all reporting an average duration of 4 years. The average duration for persistent casual partnerships was affected by the activity group of the two partners, with a much shorter duration if one of the two partners was a HA-MSM. The durations varied between 4 and 6 for a HA-MSM assortative and between 8 and 13 days for a HA-MSM disassortative partnership. The latter was in the same order of magnitude to the 12–14 days reported by Hui et al. [22], but different enough to be considered as a consistent result. Similarly, the duration varied between 251 and 299 days (between two LA-MSM individuals) which cannot be considered close to the 155 days reported by Heymans et al. [57]. Homophily rates in the literature rarely reflect on activity or risk-groups [39] and more often refer to racial, religious or socioeconomic mixing [9,13,35,36,72,73]. However, the homophily rates reported from a Dutch modeling paper were similar to our findings (75% for steady and 60% for casual partnerships) [39].

Many studies have mentioned in their limitations the lack of available high-quality social network and behavioral parameters [8,16,17,18,26,29,32,34,35,40,41,43,45]. In this paper, we present an alternative approach to estimate underlying networks, even with the lack of high-quality social network parameters. Although the focus of this paper is the structure of the underlying sexual network among Belgian participants of EMIS-2017, the same methodology could be applied to the other countries where EMIS-2017 or similar data exist. Such an experiment, would allow researchers to estimate partnership durations and homophily rates in their respective networks or highlight the areas that could prohibit such an effort.

Although the scenario defining individuals reporting more than 15 partners in a period of 12 months as the higher-activity group gave a better fit between the simulated and observed number of partners, there is still such a visible mismatch between simulated and observed numbers of cumulative partners that one could argue against the validity of the results. Both the nature of the data and the structure of the model could be suspect in our case. The EMIS-2017 data were collected among individuals using dating apps or other social media, likely yielding biased results towards the more outgoing MSM. However, since it was an anonymous and online survey, it is unlikely that the reported information suffered from desirability bias. In our effort to match the observed statistics, the model gives the best possible output to that end, though the fit is imperfect. The second factor for the mismatch is the specification of the model, both in terms of information needed to specify the mixing mechanism among MSM in our network and in terms of the definition of the sub-groups. Unfortunately, more detailed information regarding the partnership formation were not available in the EMIS-2017 dataset. More comprehensive surveys would be required to highlight the mixing mechanisms among MSM, taking into account cultural and behavioral differences among countries. In our study, the MSM population was divided into two groups, representing different levels of sexual activity, using three different definitions that have been previously used in modeling studies. Both the number of activity groups and the definition of each group drastically affect the structure of the assumed underlying network. The mismatch between the observed and simulated data, especially in the casual and one-off partnerships, might indicate the presence of more than two sub-groups in the population, which should be investigated further in future studies. Such attempts, though, could be challenging since the number of groups is uncertain and could differ among countries.

The other main limitation of our model is paradoxically the same that we were trying to rectify. Although the EMIS 2017 data are the most detailed pan-European dataset reporting MSM behavior to our knowledge, many of the parameters in the model regarding behavioral characteristics were deduced by making assumptions or by combining information in the available dataset. The need for additional high-quality data of sexual behavior that includes the behavior of the study participants and their partners remains high, especially in a public health setting where transmission and social network modeling is becoming more and more frequent. Although the current study tries to provide viable parameters for the network structure, it fails to link how these findings could be translated into the transmission and epidemiology of CT and NG in the MSM population. Several important aspects affecting STI transmission, such as the type and frequency of intercourse, condom use behaviors and the perception of the risk during oral sex, which are necessary for modeling STI transmission, were not included in the current study. Further research is required to describe and explain how the network structure affects STI epidemiology.

Notwithstanding this limitation, our study has two important findings. First, we can estimate relatable partnership durations for all three partnership types in our network in order to be able to re-create a well-defined behavioral statistic, such as the total number of partners. Second, we calculated that the ratio of homophily rates between the two activity groups is constant and can be quantified using information from the assumed underlying network. Although the proposed methodology has been regularly used in the calibration phase of modeling studies, to the best of our knowledge, it has not been used for estimating the underlying network.

The majority of the studies that provide information on model parameters come from cross-sectional or cohort studies [53,54,55,56,57]. A few studies collecting egocentric data have been published recently [74,75,76]. Egocentric data are collected by members of the population who share information about themselves (ego) and about other members with whom they interact (alters). These studies have been designed to produce parameters to inform models, thus making them the most suitable type of studies. A long list of scientists have reported a lack of good quality data which could inform transmission models [26,28,43,45]. We would like to join this call for the design and conduct of studies to inform parameters of transmission models and prove or disprove the estimated parameters from our study. While several surveys have aimed to map the epidemiological and behavioral characteristics of MSM populations, few surveys have collected the type of data that are required to inform more complex models, such as egocentric data [74,75,76]. Meanwhile, survey fatigue may hamper participation in sexual contact surveys. We recommend to perform less frequent, but more broad and in-depth surveys, targeted at collecting the data that are required to parameterize individual-based or network models.

## Figures and Tables

**Table 1:** Target and produced statistics for number of partners in the past 12 months, overall and by category.

	Target Statistics [71]		Estimated Statistics	
Number of Partners in the Past 12 Months	Mean (SD)	Median (IQR)	Mean (SD)	Median (IQR)
Overall	15.8 (36.60)	5 (2–15)	16.8 (6.00)	6 (2–19)
11–20	16.4 (3.30)	15 (14–20)	16.4 (2.59)	16 (15–19)
21–30	27.8 (2.80)	30 (25–30)	27.8 (2.29)	29 (26–30)
31–40	38.6 (2.40)	40 (37–40)	38.3 (1.63)	39 (37–40)
41–50	49.6 (1.50)	50 (50–50)	49.1 (1.01)	49 (49–50)
More than 50	128.2 (98.10)	100 (75–150)	129.0 (87.50)	99.5 (55.25–172.50)

**Table 2:** Final estimates for partnership duration and homophily rates based on model calibration.

		Prior	Weighted Mean (95% CI)
PrEP eligibility	Average duration of steady partnerships (in days)	Uniform (800, 2000)	1409 (1357–1462)
	Average duration of casual partnerships between (in days):		
	− Two HA-MSM	Uniform (4, 15)	6 (6–6)
	− Two LA-MSM	Uniform (15, 500)	251 (223–279)
	− An LA- and a HA-MSM	Uniform (10, 30)	13 (12–13)
	Homophily rates		
	− Steady HA-MSM	Uniform (0.55, 1)	0.79 (0.76–0.81)
	− Steady LA-MSM	Calculated from network	0.90
	− Casual HA-MSM	Uniform (0.45, 1)	0.65 (0.65–0.64)
	− Casual LA-MSM	Calculated from network	0.58
	− One-off HA-MSM	Uniform (0.45, 1)	0.99 (0.98–0.99)
	− One-off LA-MSM	Calculated from network	0.99
>15 partners	Average duration of steady partnerships (in days)	Uniform (800, 2000)	1065 (1031–1099)
	Average duration of casual partnerships between (in days):		
	− Two HA-MSM	Uniform (3, 100)	4 (4–4)
	− Two LA-MSM	Uniform (15, 500)	299 (272–326)
	− An LA- and a HA-MSM	Uniform (5, 100)	11 (11–11)
	− Homophily rates		
	− Steady HA-MSM	Uniform (0.55, 1)	0.75 (0.73–0.78)
	− Steady LA-MSM	Calculated from network	0.90
	− Casual HA-MSM	Uniform (0.60, 1)	0.68 (0.67–0.69)
	− Casual LA-MSM	Calculated from network	0.35
	− One-off HA-MSM	Uniform (0.45, 1)	0.98 (0.98–0.99)
	− One-off LA-MSM	Calculated from network	0.98
>15 casual partners	Average duration of steady partnerships (in days)	Uniform (800, 2000)	1314 (1260–1367)
	Average duration of casual partnerships between (in days):		
	− Two HA-MSM	Uniform (3, 100)	4 (4–4)
	− Two LA-MSM	Uniform (15, 500)	266 (244–289)
	− An LA- and a HA-MSM	Uniform (5, 100)	8 (8–9)
	Homophily rates		
	− Steady HA-MSM	Uniform (0.55, 1)	0.77 (0.74–0.79)
	− Steady LA-MSM	Calculated from network	0.92
	− Casual HA-MSM	Uniform (0.45, 1)	0.69 (0.68–0.7)
	− Casual LA-MSM	Calculated from network	0.48
	− One-off HA-MSM	Uniform (0.45, 1)	0.98 (0.98–0.99)
	− One-off LA-MSM	Calculated from network	0.98

## Data Availability

The EMIS-2017 data that were used to parametrize the model in this paper are available upon request to the EMIS-2017 consortium (https://www.emis-project.eu/, accessed on 29 April 2024).

## Supplementary Materials

The following supporting information can be downloaded at: https://www.mdpi.com/article/10.3390/idr16030032/s1, Figure S1: Observed and simulated cumulative casual sex partner distribution (12 months); Table S1: Partnership formation probabilities for steady and casual partnerships stratified by partnership group, Table S2: Partnership formation probabilities for steady and persistent casual partnerships stratified by partnership group, Table S3: Partnership distribution among persons with an one-off partner stratified by partnership group, Table S4: One-off partnership parameters, Table S5: Homophily rates parameters, Table S6: Sex act rate probabilities per partnership type, Table S7: Combination of sex acts and probabilities, Table S8: Behavioral characteristics per activity group.
