# Supplementary material for: Estimating Partnership Duration among MSM in Belgium—A Modeling Study
Source: Infect Dis Rep. 2024 May 6;16(3):435–47. doi: 10.3390/idr16030032 (PMC11130929; doi:10.3390/idr16030032)
Supplement: Supplementary file 1 [file idr-16-00032-s001.zip › idr-2868174-supplementary.pdf]

Supplementary Material

1. General description

The current document describes a network-based model for estimating the duration of partnerships among men who have sex with men (MSM) and the modules accompanying it. The whole project is developed as an extension of the EpiModel platform (https://www.epimodel.org/). The current project uses the statistical framework of separable temporal exponential-family random graph models (STERGMs) to fit and simulate dynamic networks[1,2]. The framework has been extensively described in Goodreau et al[3] and Jenness et al[4,5], as well as in the EpiModel page (www.epimodel.org). Individual attributes related to this partnership formation are stored and updated in discrete time over the course of each epidemic simulation. Statistical software R v.4.3.0 [6] and packages Epimodel[7] v2.4.0 were used for the programming of this model.

2. Data

Data from the Belgian-based participants of the European MSM Internet Survey (EMIS) 2017 were the primary data source for network structure and behavioral parameters used in the model. EMIS-2017 was an anonymous, self-administered online survey conducted in 50 countries and 33 languages. Participants were recruited from dating and other social networking websites targeting MSM. The methods of EMIS-2017 have been described in detail elsewhere[8]. In total 3038 persons based in Belgium filled in the study questionnaire, with 2763 reporting answers without any inconsistencies. Data recorded in the EMIS dataset included information on the participants' partnership status, behavioral and epidemiological characteristics.

The term "steady partners" was used to describe husbands, boyfriends or the partners with whom EMIS participants would not identify themselves as single. Persistent casual partners were the partners that would not qualify as steady, but the EMIS participants would have sex with more than one time. One-off partners were casual partners whom EMIS participants would only have sex with once.

3. Model structure

3.1. Population groups and model initialization

Individuals in the network were categorized into two groups, according to their sexual activity level to account for systematic behavioral patterns within the MSM population. Three different definitions were used to define high-activity behavior. Eligibility to receive PrEP, individuals reporting more than 15 partners over 12 months and individuals reporting more than 15 casual partners over 12 months were all used , as a proxy to define MSM with a higher-risk sex behavior. In Belgium, eligibility for PrEP includes being above 18 years old, being HIV negative and fulfilling one of the following criteria:

- Anal sex without a condom with at least two partners in the previous 6 months
- Multiple sexually transmitted infections (STIs) in the previous 12 months
- Post-exposure prophylaxis (PEP) multiple times in the previous 12 months
- Use of psychotropic substances (drugs) during sexual activities

Individuals were considered to belong to the higher-activity group (HA-MSM) if they were either taking PrEP, or if they were eligible to participate in the PrEP program. Similarly participants reporting more than 15 (steady and non-steady) partners over a period of 12 months or participants reporting more than 15 casual partners (non-steady partners) were classified as HA-MSM in their

respective scenarios. Those not classified as HA-MSM, were classified in the lower-activity group (LA-MSM).

According to the EMIS dataset, 34.6% classified as HA-MSM. This estimate is similar to previously published studies from Belgium and Europe[9–11]. The two groups were assumed to have different sex act rates and condom use and screening behaviors.

We initialized a population of 10,000 Belgian MSM and randomly allocated them to the two groups according to the estimated proportions.

3.2. Partnership types

The model consists of three parallel, interacting networks representing steady, persistent casual and one-off (one-night stand) partnerships. As steady partners, the EMIS questionnaire specified those who are a lover or spouse that means that participants identified as "not single". All other partners were coded as casual partners. Among casual partners, the EMIS questionnaire established whether participants have had sex with them before (once, or more than once). Those who reported partners that they had not have sex before, were coded as one-off partners (one-night stands), while the remaining as persistent casual partners. The three parallel networks contained the same set of persons who were further distinguished into two groups (LA- and HA-MSM) as described above.

3.3. Partnership formation and dissolution

The formation of partnerships in all three networks (steady, casual, and one-off) was governed by similar formation equations, in order to be able to preserve the distinct behavioral characteristics linked to each partnership type. The formation of steady (and casual) partnerships was associated with the number of total number of steady (casual) partnerships currently in the network, the proportion of concordant partnerships (HA-MSM with HA-MSM, or LA-MSM with LA-MSM) different for each group, the proportion of individuals with concurrent partners (2 or more active partnerships simultaneously) and their status regarding casual (steady) partners (proportion of individuals with 0, 1, or more than 1 casual (steady) partners). The formation of one-off partnerships depended on the total number of one-off partnerships, the proportion of concordant partnerships (different for each group), the proportion of individuals with 0, 1 or more than 1 steady partners, and the proportion of individuals with 0, 1, or more than 1 casual partners. All those characteristics were targeted to match observed statistics from the EMIS 2017 dataset for each group and partnership type and the underlying network was fitted so that they would be held constant over time.

The number of ongoing partnerships (steady and persistent casual) and their combinations were estimated using the EMIS 2017 dataset. We allowed each individual in the model to have zero, one or more than one steady partner and zero, one or more than one casual partners at any given time step.

The distribution of steady and persistent casual partnerships by group is presented in
Table S1.

Table S1: Partnership formation probabilities for steady and casual partnerships stratified by partnership group

			**All Casual partners**		
**PrEP eligibility**	Lower-activity MSM	Steady partners	0	1	>1
		0	51.51 %	4.35 %	1.03 %
		1	32.13 %	7.25 %	1.99 %
		> 1	1.27 %	0.30 %	0.18 %

	Higher-activity MSM	Steady partners			
		0	49.95 %	9.10 %	4.23 %
		1	20.59 %	10.51 %	3.36 %
		> 1	1.19 %	0.54 %	0.54 %
**More than 15 partners**	Lower-activity MSM	Steady partners	0	1	>1
		0	54.13 %	4.07 %	0.46 %
		1	32.05 %	6.94 %	0.86 %
		> 1	1.26 %	0.17 %	0.06 %
	Higher-activity MSM	Steady partners			
		0	43.73 %	10.40 %	5.89 %
		1	19.05 %	12.03 %	6.14 %
		> 1	1.00 %	0.88 %	0.88 %
**More than 15 casual partners**	Lower-activity MSM	Steady partners	0	1	>1
		0	53.12 %	3.92 %	0.5 %
		1	32.28 %	7.52 %	1.05 %
		> 1	1.22 %	0.22 %	0.17 %
	Higher-activity MSM	Steady partners			
		0	45.29 %	11.32 %	6.28 %
		1	17.33 %	11.05 %	6.14 %
		> 1	1.09 %	0.82 %	0.68 %

Table S2: Partnership formation probabilities for steady and persistent casual partnerships stratified by partnership group

			**Persistent casual partners**		
**PreP eligibility**	Lower-activity MSM	Steady partners	0	1	>1
		0	54.47%	2.11 %	0.30 %
		1	37.50 %	3.02 %	0.84 %
		> 1	1.57 %	0 %	0.18 %
	Higher-activity MSM	Steady partners			
		0	54.71 %	6.39 %	2.17 %
		1	27.52 %	4.98 %	1.95 %
		> 1	1.30 %	0.54 %	0.43 %
**More than 15 partners**	Lower-activity MSM	Steady partners	0	1	>1
		0	56.54 %	1.95 %	0.17 %
		1	37.04 %	2.29 %	0.52 %
		> 1	1.38 %	0.06 %	0.06 %
	Higher-activity MSM	Steady partners			
		0	50.00 %	7.27 %	2.76 %
		1	27.32 %	7.02 %	2.88 %
		> 1	1.50 %	0.50 %	0.75 %
**More than 15 casual partners**	Lower-activity MSM	Steady partners	0	1	>1

		0	55.44 %	1.88 %	0.22 %
		1	37.76 %	2.38 %	0.72 %
		> 1	1.38 %	0.06 %	0.17 %
	Higher-activity MSM	Steady partners			
		0	52.11 %	7.91 %	2.86 %
		1	24.68 %	7.23 %	2.59 %
		> 1	1.50 %	0.55 %	0.55 %

Table S3: Partnership distribution among persons with an one-off partner stratified by partnership group

PreP eligibility	Lower-activity MSM	Steady partners	0	1	> 1
		Steady partners	3.38 %	5.98 %	0.28 %
		Persistent casual	7.97 %	1.83 %	0.00 %
	Higher-activity MSM	Steady partners	6.58 %	8.05 %	0.21 %
		Persistent casual	11.70 %	3.55 %	0.00 %
**More than 15 partners**	Lower-activity MSM	Steady partners	2.43 %	4.80 %	0.10 %
		Persistent casual	6.91 %	0.72 %	0.00 %
	Higher-activity MSM	Steady partners	8.57 %	10.51 %	0.57 %
		Persistent casual	13.83 %	5.94 %	0.00 %
**More than 15 casual partners**	Lower-activity MSM	Steady partners	2.34 %	5.23 %	0.15 %
		Persistent casual	7.33 %	0.70 %	0.00 %
	Higher-activity MSM	Steady partners	9.29 %	9.91 %	0.50 %
		Persistent casual	13.38 %	6.44 %	0.00 %

Table S4: One-off partnership parameters

		**Lower-activity MSM**	**Higher-activity MSM**
**Prep Eligibility**	**Proportion of one-off partnerships among persons with at least 1 casual partner**	69.14%	54.48%
	**Proportion of concurrent one-off partners**	57.89%	43.59%
**More than 15 partners**	**Proportion of one-off partnerships among persons with at least 1 casual partner**	65.49 %	58.64 %

	**Proportion of concurrent one-off partners**	51.85 %	48.60 %
**More than 15 casual partners**	**Proportion of one-off partnerships among persons with at least 1 casual partner**	64.40 %	59.04 %
	**Proportion of concurrent one-off partners**	40.00 %	52.53%

For both steady and persistent casual partnerships, there was a constant hazard of relationship dissolution, modeled as a memoryless process. For steady partnerships, we used a constant hazard depending on the number of total number of partnerships present in the network. For casual partners, the dissolution of partnerships depending on the group of the two partners in a dyad (different for the HA- and LA-MSM). The duration of one-off partnerships was set to 1 day.

3.4. Homophily in partnership formation by group

The role of homophily, or tendency of people to form sexual partnerships or other kind of social bonds with persons similar to them in terms of various characteristics has been well established in the social network literature[12–16].

We adopted the premise from the paper of Hansson et al[11] that the number of HA-individuals having LA-partners should be the same as the number of LA-individuals having a HA-partner within the same MSM population. This can be written as

$$B^{HL} = B^{LH} \text{ (1)},$$

where B^{ij} is the number of disassortative (mixed) partnerships between an individual of group i and an individual from group j, with i ≠ j, where the type of partnerships could be steady, casual, or one-off. The number of disassortative partnerships B is the product of the complementary of the proportion of assortative partnerships in the group and the total number of partnerships among individuals in group i. For example, for the HA-group,

$$B^{HL} = F_H \cdot (1 - h_{HH}) \text{ (2)},$$

where F_H = number of partnerships that HA-individuals have and h_{HH} is the proportion of assortative (H-H) partnerships (homophily). Quantity F in equation (2) could denote steady, casual or one-off partnerships. If F denotes casual partnerships, then $F_H = C_H \cdot n_H$, where C_H is the proportion of HA-individuals with casual partners and n_H is the number of HA-individuals in the population, which can be easily calculated as the product of the proportion of HA-individuals in the population (p_H) times the population size. The equations for the LA-group can be easily constructed in the same manner. By replacing each side of equation (1) we get

$$n \cdot p_H \cdot C_H \cdot (1 - h_{HH}) = n \cdot p_L \cdot C_L \cdot (1 - h_{LL})$$

which simplifies into:

$$\frac{p_H}{P_L} = \frac{C_L}{C_H} \cdot \frac{(1 - h_{LL})}{(1 - h_{HH})} \text{ (3)}$$

By replacing the estimated risk group ratio in the population and the ratio of steady, casual or one-off partnerships, we end up with an equation of the form $h_{LL} = a + b \cdot h_{HH}$, as shown in Table S5 below.

Table S5: Homophily rates parameters

**PreP eligbility**	Main	$H_{LL} = 0.44 * h_{HH} + 0.55$
	Persistent casual	$H_{LL} = 1.37 * h_{HH} – 0.37$
	One-off	$H_{LL} = 0.75 * h_{HH} + 0.25$
**More than 15 partners**	Main	$H_{LL} = 0.44 * h_{HH} + 0.56$
	Persistent casual	$H_{LL} = 2.08 * h_{HH} – 1.08$
	One-off	$H_{LL} = 1.29 * h_{HH} – 0.13$
**More than 15 casual partners**	Main	$H_{LL} = 0.35 * h_{HH} + 0.65$
	Persistent casual	$H_{LL} = 1.64 * h_{HH} – 0.64$
	One-off	$H_{LL} = 0.96 * h_{HH} + 0.04$

Homophily rate estimates were not available in the EMIS 2017 dataset, nor in the literature, thus we calibrated these parameters together with the duration parameters as described in more detail in Section 5.

4. Model processes

In summary, exponential-family random graph models (ERGMs) and their temporal extension separable temporal ERGMs (STERGMs) provide a framework for simulation of structures given a set of target statistics. The conditional probabilities of formation and dissolution of edges (partnerships) are governed by a set of equations, different for formation and dissolution and each partnership type, depend on a function of network statistics (g and a parameter vector θ). Thus, the log-odds of the formation of edges is given by an equation of the form:

$$logit\left(P\left(Y_{ij,t} = 1 | Y_{ij,t-1} = 0, Y_{ij,t}^{c}\right)\right) = \theta_R^{+'} \partial(g_R^{+}(y)),$$

where $Y_{ij,t}$ is the relational status of persons i and j at time t (1 = in relationship, 0 = not), $Y_{ij,t}^{c}$ is the network complement of i,j at time t, i.e. all relations in the network other than i,j, g(y) is vector of network statistics in each model, θ is the vector of parameters in the formation model and R is the relationship type. The formula for the log-odds of dissolution is similar to formula for formation, using different functions of network statistics and parameters.

Once the network of interest was initialized, the following steps occurred at each simulation:

At time step 1:

1. Random allocation of characteristics behavioral characteristics like condom use and screening behavior.
2. Increment time by one step

At time step > 1:

1. Update of network given the new time point in terms of creating new relationships or dissolving current ones.
2. Calculation of the number of sex acts that will occur in each active partnership.
3. Calculation of network summary statistics
4. Increment time by one step.

In this document as well as in the steady text, a time step refers to one day, a month refers to a period of 30 days, and a year is a period of 12 months or 360 days.

We simulated the model 50 times for 360 days. The merged data from all 50 simulations were used to analyze the results. The computational resources and services used in this work were provided by the HPC core facility CalcUA of the Universiteit Antwerpen, and VSC (Flemish Supercomputer Center), funded by the Research Foundation - Flanders (FWO) and the Flemish Government.

4.1. Sex acts

At each time step of the simulation, a list of active partnerships and their respective type (steady, casual or one-off) was created based on the current composition of the network, which is called an "edge-list". Partnerships were considered as active at a particular time step if the terminus of the partnership was equal to or greater than the current time step. Based on the type of relationship and the group combination of the partnership, the number of sex acts that occurred between the two partners on a given time step was calculated by random draws from a Binomial distribution with a success probability depending on the type of partnership and the group of the two partners, as shown in the Table S6 below. The mean sex act rate for mixed partnerships (a higher- and a lower-activity partner) was not available in the primary data source, and the average of the act rates of the two homogenous partnership combinations was assumed instead. The act rate for one-off partnerships was set to 100%.

Table S6: Sex act rate probabilities per partnership type

		**Steady partnerships**			**Casual partnerships**	
**PrEP eligibility**		Lower-activity MSM	Higher-activity MSM		Lower-activity MSM	Higher-activity MSM
	Lower-activity MSM	27.94 %		Lower-activity MSM	67.72 %	
	Higher-activity MSM	34.13 %	40.32 %	Higher-activity MSM	81.67 %	95.62 %
**More than 15 partners**		Lower-activity MSM	Higher-activity MSM		Lower-activity MSM	Higher-activity MSM
	Lower-activity MSM	25.77 %		Lower-activity MSM	68.66 %	

	Higher-activity MSM	37.73 %	49.69 %	Higher-activity MSM	83.98 %	99.29 %
**More than 15 casual partners**		Lower-activity MSM	Higher-activity MSM		Lower-activity MSM	Higher-activity MSM
	Lower-activity MSM	26.90 %		Lower-activity MSM	69.64 %	
	Higher-activity MSM	37.44 %	47.97 %	Higher-activity MSM	84.59 (	99.53 %

The pairs where 0 sex acts were predicted were removed from the edge-list, and the final edge-list contained the IDs of the two partners, the type of relationship (steady/casual) on a given time step.

Each sex act contained a combination of six sex types: oral sex (between urethra and pharynx), oro-anal (between pharynx and rectum) and anal sex (between urethra and rectum), each of which could be either insertive or receptive. As insertive and receptive we define the sex role of the partners in each sex act. Without loss of generality, the first partner in the edge list was always assumed to be reporting the sex act, thus, the insertive/receptive sex act will reflect that person's sex role. For example, if insertive oral sex was reported, then the anatomical sites involved was the first person's urethra and the second person's pharynx. Each combination of sex types was selected randomly based on the frequency of sex act combinations as reported in the EMIS-2017 (Table S7). Some combinations of sex types in the published table returned zero probability, which would be unrealistic at the population level. Thus, the probabilities for each combination of sex type were adjusted to correct for this shortcoming of the data. Half of the smallest observed probability (0.02%) was arbitrarily assigned to all combinations with a zero probability in the dataset. The remaining probabilities were adjusted using the assumption of a fixed ratio between the adjusted and original probabilities, in order to ensure the sum of all probabilities equals 1.

Table S7: Combination of sex acts and probabilities

**Oral sex**		**Oro-anal sex**		**Anal sex**		**Probability**	**Adjusted probability**
Insertive	Receptive	Insertive	Receptive	Insertive	Receptive		
0	0	0	0	0	1	0.0138	0.0137
0	0	0	0	1	0	0.0133	0.0133
0	0	0	0	1	1	0.0014	0.0014
0	0	0	1	0	0	0.0014	0.0014
0	0	0	1	0	1	0.0033	0.0033
0	0	0	1	1	0	0.0000	0.0002
0	0	0	1	1	1	0.0014	0.0014
0	0	1	0	0	0	0.0009	0.0009
0	0	1	0	0	1	0.0014	0.0014
0	0	1	0	1	0	0.0009	0.0009

0	0	1	0	1	1	0.0000	0.0002
0	0	1	1	0	0	0.0000	0.0002
0	0	1	1	0	1	0.0000	0.0002
0	0	1	1	1	0	0.0009	0.0009
0	0	1	1	1	1	0.0000	0.0002
0	1	0	0	0	0	0.0475	0.0474
0	1	0	0	0	1	0.0456	0.0455
0	1	0	0	1	0	0.0038	0.0038
0	1	0	0	1	1	0.0014	0.0014
0	1	0	1	0	0	0.0024	0.0024
0	1	0	1	0	1	0.0214	0.0213
0	1	0	1	1	0	0.0005	0.0005
0	1	0	1	1	1	0.0014	0.0014
0	1	1	0	0	0	0.0081	0.0081
0	1	1	0	0	1	0.0128	0.0128
0	1	1	0	1	0	0.0033	0.0033
0	1	1	0	1	1	0.0019	0.0019
0	1	1	1	0	0	0.0005	0.0005
0	1	1	1	0	1	0.0071	0.0071
0	1	1	1	1	0	0.0009	0.0009
0	1	1	1	1	1	0.0009	0.0009
1	0	0	0	0	0	0.0328	0.0327
1	0	0	0	0	1	0.0028	0.0028
1	0	0	0	1	0	0.0394	0.0393
1	0	0	0	1	1	0.0005	0.0005
1	0	0	1	0	0	0.0085	0.0085
1	0	0	1	0	1	0.0043	0.0043
1	0	0	1	1	0	0.0047	0.0047
1	0	0	1	1	1	0.0009	0.0009
1	0	1	0	0	0	0.0033	0.0033
1	0	1	0	0	1	0.0005	0.0005
1	0	1	0	1	0	0.0109	0.0109
1	0	1	0	1	1	0.0000	0.0002
1	0	1	1	0	0	0.0005	0.0005
1	0	1	1	0	1	0.0000	0.0002
1	0	1	1	1	0	0.0024	0.0024
1	0	1	1	1	1	0.0009	0.0009
1	1	0	0	0	0	0.1201	0.1199
1	1	0	0	0	1	0.0736	0.0735
1	1	0	0	1	0	0.0689	0.0687
1	1	0	0	1	1	0.0190	0.0190
1	1	0	1	0	0	0.0190	0.0190
1	1	0	1	0	1	0.0802	0.0801
1	1	0	1	1	0	0.0123	0.0123
1	1	0	1	1	1	0.0190	0.0190
1	1	1	0	0	0	0.0171	0.0171
1	1	1	0	0	1	0.0123	0.0123
1	1	1	0	1	0	0.0632	0.0630

1	1	1	0	1	1	0.0104	0.0104
1	1	1	1	0	0	0.0157	0.0156
1	1	1	1	0	1	0.0456	0.0455
1	1	1	1	1	0	0.0385	0.0384
1	1	1	1	1	1	0.0741	0.0740

4.2. Behavioral characteristics

EMIS-2017participants were classified in regard to sex role, as either exclusively insertive (active), receptive (passive) or as versatile, if they reported being both insertive and receptive in their last sex act.

Condom use and STI screening behavior were included in the population at the network initiation step. A proportion of the population in the network were stochastically assigned during network initiation as consistent condom users with all their casual partners calculated from EMIS-2017 dataset.

The European STI screening guidelines for MSM recommend at least annual 3-site screening[17,18], with PrEP users attending 3-monthly screening visits. However, the Belgian EMIS-2017 participants, reported significantly lower percentages, varying across the three definitions. The majority reported only providing samples from the urethra (urine sample or urethral swab) and only a small fraction provided samples only a pharyngeal or rectal swab.

Regarding the screening frequency, almost 30% of the LA-MSM and 10% of the HA-MSM of Belgian EMIS-2017 participants reported that they have never screened for STIs in all three definitions. The percentages of LA- and HA-MSM who reported that had an STI test during the previous 6 or 12 months, differed among the three definitions, but not considerably..

Table S8: Behavioral characteristics per activity group

Activity group	Behavioural characteristic	Lower-activity MSM	Higher-activity MSM
**PrEP eligibility**	**Sex role: Insertive**	**38.85 %**	**34.43 %**
	**Receptive**	**48.15 %**	**42.03 %**
	**Versatile**	**13.00 %**	**23.54 %**
	**Consistent condom users**	**67.72 %**	**8.06 %**
	**Never screen for STIs**	**32.30 %**	**10.72 %**
	**STI screening in the previous 12 months**	**67.40 %**	**89.80 %**
	**STI screening in the previous 6 months**	**46.38 %**	**77.79 %**
	**Screening sites: Urethra only**	**75.54 %**	**49.24 %**
	**Pharynx and/or rectum**	**2.79 %**	**2.36 %**
	**Pharynx, Urethra and rectum**	**21.67 %**	**48.40 %**

**More than 15 partners**	**Sex role: Insertive**	**35.52 %**	**37.72 %**
	**Receptive**	**49.20 %**	**39.28 %**
	**Versatile**	**15.29 %**	**23.00 %**
	**Consistent condom users**	**45.74 %**	**29.46 %**
	**Never screen for STIs**	**31.61 %**	**9.55 %**
	**STI screening in the previous 12 months**	**70.56 %**	**86.97 %**
	**STI screening in the previous 6 months**	**50.84 %**	**73.39 %**
	**Screening sites: Urethra only**	**49.90 %**	**70.55 %**
	**Pharynx and/or rectum**	**3.07 %**	**2.00 %**
	**Pharynx, Urethra and rectum**	**47.03 %**	**27.45 %**
**More than 15 casual partners**	**Sex role: Insertive**	**35.79 %**	**37.48 %**
	**Receptive**	**48.16 %**	**40.03 %**
	**Versatile**	**16.05 %**	**22.49 %**
	**Consistent condom users**	**44.87 %**	**29.53 %**
	**Never screen for STIs**	**31.03 %**	**9.05 %**
	**STI screening in the previous 12 months**	**71.21 %**	**87.07 %**
	**STI screening in the previous 6 months**	**51.58 %**	**73.85 %**
	**Screening sites: Urethra only**	**75.54 %**	**49.24 %**
	**Pharynx and/or rectum**	**2.79 %**	**2.36 %**
	**Pharynx, Urethra and rectum**	**21.67 %**	**48.40 %**

5. Parameter estimation

5.1. Estimation methods

For all parameters that were not available in the EMIS-2017 data, we used approximate Bayesian computation with sequential Monte Carlo sampling (ABC-SMC) to estimate those parameters. The method of ABC-SMC has been extensively described in the literature[19–22] and is particularly useful in cases where the likelihood is not tractable. The ABC-SMC method returns estimations for the posterior distribution of the parameters of interest after defining prior distributions. The Lenormand method of the EasyABC package[23] (Version 1.5) was used for the parameter estimation.

5.1.1. Network calibration: Partnership duration and homophily

Estimates for the partnership durations among MSM for each partnership type and/or risk group were not available in the EMIS questionnaire. In Buyze et al[24], the average partnership duration was 2318 days for steady and 62 days for casual, while in Reitsema et al[25] the average duration for steady partnerships was 1355 days and for casual partnerships 155 days. Neither paper made a distinction for activity-group or for one-off partnerships. To evaluate these parameter estimates from literature, we opted to use the cumulative number of partners per partnership type and group over a period of 12 months as a measure of goodness-of-fit for our model. The total number of steady and casual partners over a period of 12 months is reported in the EMIS 2017 dataset, but as a categorical

variable: 0, 1, 2, …, 9, or 10 or more for steady partners and 0, 1, 2, …, 10, 11-20, 21-30, 31-40, 41-50, or more than 50 for casual. Assuming at most 12 steady partners over 12 months, we used the methodology and statistics mentioned in Mendez-Lopez et al[26], to build an algorithm to assign integer values to the number of total partners that match the mean (standard deviation) and median (interquartile range) for each category level and overall. With the estimated number of total partners, we could calculate the cumulative number of steady and casual partners per risk-group over 12 months.

We opted to calibrate our model, using a data-driven approach, in terms of partnership durations and homophily rates. A model was simulated for one year with 20 simulations and the distribution of cumulative steady, casual, and one-off partners was compared to those reported among EMIS-2017 participants, for each group, in terms of mean, median and interquartile range. Since the process for estimating the parameters was too computationally intensive using the ABC-SMC method, we reduced the parameter space by dividing the ranges for each of the parameters into smaller sections and calculating the absolute error between the produced location and spread statistics to the observed ones. This process was repeated a number of times, with the range and step size being refined until the resulting distribution was similar to the reported one. Once the parameter space was deemed sufficiently optimal, we used ABC-SMC with the range of the last set of parameters as the priors, to obtain the final estimates for the partnership durations.

Using this information we calibrated the partnership duration parameters in our model to reproduce the distribution of total unique partners over a 12-month period as reported in EMIS 2017. We used Approximate Bayesian Computation (ABC) with the number of sex partners as targets.

6. Additional results

The analysis described in the main manuscript was repeated using categorical variables for the number of partners produced by simulations and the observed frequencies reported in the EMIS-2017 dataset, both overall and by activity group. The results are similar to the ones in the main manuscript with the definition of more than 15 casual partners giving a better fit to the observed numbers of partners.

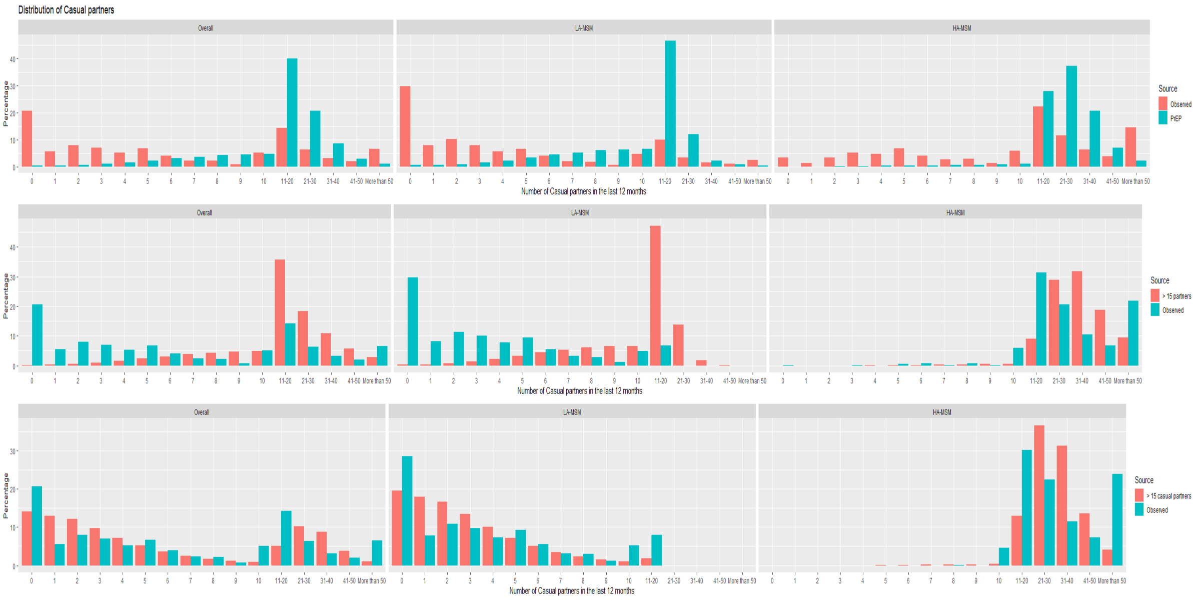

Figure S1: Observed and simulated cumulative casual sex partner distribution (12 months).

7. Bibliography

1. Krivitsky PN, Handcock MS. A separable model for dynamic networks. *J R Stat Soc Ser B Stat Methodol*. 2014;76(1):29-46. doi:10.1111/rssb.12014
2. Hunter DR, Handcock MS, Butts CT, Goodreau SM, Morris M. ergm: A package to fit, simulate and diagnose exponential-family models for networks. *J Stat Softw*. 2008;24(3):1-29. doi:10.18637/jss.v024.i03
3. Goodreau SM, Carnegie NB, Vittinghoff E, et al. What Drives the US and Peruvian HIV Epidemics in Men Who Have Sex with Men (MSM)? *PLoS One*. 2012;7(11). doi:10.1371/journal.pone.0050522
4. Jenness SM, Goodreau SM, Rosenberg E, et al. Impact of the centers for disease control's HIV preexposure prophylaxis guidelines for men who have sex with men in the United States. *J Infect Dis*. 2016;214(12):1800-1807. doi:10.1093/infdis/jiw223
5. Jenness SM, Weiss KM, Goodreau SM, et al. Incidence of gonorrhea and chlamydia following human immunodeficiency virus preexposure prophylaxis among men who have sex with men: A modeling study. *Clin Infect Dis*. 2017;65(5):712-718. doi:10.1093/cid/cix439
6. R Core Team (2017). R: A language and environment for statistical computing. R Foundation for Statistical Computing, Vienna, Austria. URL https://www.R-project.org/.
7. Jenness SM, Goodreau SM, Morris M. Epimodel: An R package for mathematical modeling of infectious disease over networks. *J Stat Softw*. 2018;84(8). doi:10.18637/jss.v084.i08
8. Weatherburn P, Hickson F, Reid DS, Marcus U, Schmidt AJ. European Men-Who-Have-Sex-With-Men Internet Survey (EMIS-2017): Design and Methods. *Sex Res Soc Policy*. 2020;17(4):543-557. doi:10.1007/s13178-019-00413-0
9. Buffel V, Reyniers T, Masquillier C, et al. Awareness of, Willingness to Take PrEP and Its Actual Use Among Belgian MSM at High Risk of HIV Infection: Secondary Analysis of the Belgian European MSM Internet Survey. *AIDS Behav*. 2022;26(6):1793-1807. doi:10.1007/s10461-021-03526-z
10. Sherriff NS, Jones AM, Mirandola M, et al. Factors related to condomless anal intercourse between men who have sex with men: Results from a European bio-behavioural survey. *J Public Heal (United Kingdom)*. 2020;42(2):1-13. doi:10.1093/pubmed/fdz052
11. Hansson D, Strömdahl S, Leung KY, Britton T. *Introducing Pre-Exposure Prophylaxis to Prevent HIV Acquisition among Men Who Have Sex with Men in Sweden: Insights from a Mathematical Pair Formation Model*. Vol 10.; 2020. doi:10.1136/bmjopen-2019-033852
12. Krivitsky PN, Handcock MS, Morris M. Adjusting for network size and composition effects in exponential-family random graph models. *Stat Methodol*. 2011;8(4):319-339. doi:10.1016/j.stamet.2011.01.005
13. Liu C, Lu X. Network evolution of a large online MSM dating community: 2005–2018. *Int J Environ Res Public Health*. 2019;16(22). doi:10.3390/ijerph16224322
14. Currarini S, Vega Redondo F. A Simple Model of Homophily in Social Networks Department of Economics A Simple Model of Homophily in Social Networks. 2011;(16).
15. Cai M, Huang G, Kretzschmar ME, Chen X, Lu X. Extremely Low Reciprocity and Strong Homophily in the World Largest MSM Social Network. *IEEE Trans Netw Sci Eng*. 2021;8(3):2279-2287. doi:10.1109/TNSE.2021.3085984
16. Murase Y, Jo HH, Török J, Kertész J, Kaski K. Structural transition in social networks: The role of homophily. *Sci Rep*. 2019;9(1):1-8. doi:10.1038/s41598-019-40990-z
17. Lanjouw E, Ouburg S, de Vries HJ, Stary A, Radcliffe K, Unemo M. 2015 European guideline on the management of Chlamydia trachomatis infections. *Int J STD AIDS*. 2016;27(5):333-348. doi:10.1177/0956462415618837
18. Pharris A, Amato AJ, Carrillo-Santisteve P, et al. *HIV and STI Prevention among Men Who Have Sex with Men HIV and STI Prevention among Men Who Have Sex with Men-Gauci with*

Technical Input From.; 2015. https://ecdc.europa.eu/sites/portal/files/media/en/publications/Publications/hiv-sti-prevention-among-men-who-have-sex-with-men-guidance.pdf
19. Toni T, Welch D, Strelkowa N, Ipsen A, Stumpf MPH. Approximate Bayesian computation scheme for parameter inference and model selection in dynamical systems. *J R Soc Interface*. 2009;6(31):187-202. doi:10.1098/rsif.2008.0172
20. Held L, Hens N, O'Neil P, Wallinga J. *Handbook of Infectious Disease Data Analysis*. Chapman and Hall/CRC; 2020. doi:https://doi.org/10.1201/9781315222912
21. Beaumont MA, Cornuet JM, Marin JM, Robert CP. Adaptive approximate Bayesian computation. *Biometrika*. 2009;96(4):983-990. doi:10.1093/biomet/asp052
22. Sisson SA, Fan Y, Tanaka MM. Sequential Monte Carlo without likelihoods (Proceedings of the National Academy of Sciences of the United States of America (2007) 104, 6, (1760-1765) DOI:10.1073/pnas.0607208104). *Proc Natl Acad Sci U S A*. 2009;106(39):16889. doi:10.1073/pnas.0908847106
23. Jabot F, Faure T, Dumoulin N. EasyABC: Efficient Approximate Bayesian Computation Sampling Schemes. Published online 2015.
24. Buyze J, Vanden Berghe W, Hens N, Kenyon C. Current levels of gonorrhoea screening in MSM in Belgium may have little effect on prevalence: A modelling study. *Epidemiol Infect*. 2018;146(3):333-338. doi:10.1017/S0950268818000092
25. Reitsema M, Heijne J, Visser M, et al. Impact of frequent testing on the transmission of HIV and N. gonorrhoeae among men who have sex with men: A mathematical modelling study. *Sex Transm Infect*. 2020;96(5):361-367. doi:10.1136/sextrans-2018-053943
26. Mendez-Lopez A, Hickson F, Jansen K, et al. What is the empirical basis for converting banded ordinal data on numbers of sex partners among MSM into a continuous scale level variable? A secondary analysis of 13 surveys across 17 countries. *BMC Med Res Methodol*. 2022;22(1):1-7. doi:10.1186/s12874-021-01483-8
